# An immunoinformatics assessment of the cancer testis antigen, DDX53, as a potential early esophageal cancer antigen

**DOI:** 10.18632/oncoscience.590

**Published:** 2023-11-10

**Authors:** Peter Cheng, Konrad J. Cios, Mallika Varkhedi, Vayda R. Barker, Michelle Yeagley, Andrea Chobrutskiy, Boris I. Chobrutskiy, George Blanck

**Affiliations:** ^1^Department of Molecular Medicine, Morsani College of Medicine, University of South Florida, Tampa, Florida (FL) 33612, USA; ^2^Department of Pediatrics, Oregon Health and Science University Hospital, Portland, Oregon (OR) 97239, USA; ^3^Department of Internal Medicine, Oregon Health and Science University Hospital, Portland, Oregon (OR) 97239, USA; ^4^Department of Immunology, H. Lee Moffitt Cancer Center and Research Institute, Tampa, Florida (FL) 33612, USA

**Keywords:** adaptive immune receptor recombinations, complementarity determining region-3, esophageal cancer survival rates, cancer testis antigens

## Abstract

T-lymphocytes have been implicated in facilitating a pro-inflammatory, pro-tumorigenic microenvironment that worsens prognosis for esophageal carcinoma (ESCA). In this study, we identified tumor resident, T-cell receptor (TCR) complementarity determining region-3 (CDR3) amino acid sequences and employed an algorithm particularly suited to the big data setting to evaluate TCR CDR3-cancer testis antigen (CTA) chemical complementarities. Chemical complementarity of the ESCA TCR CDR3s and the cancer testis antigen DDX53 represented a disease-free survival (DFS) distinction, whereby the upper fiftieth percentile complementarity group correlated with worse DFS. The high TCR CDR3-DDX53 complementarity group also represented a greater proportion of tumor samples lacking DDX53 expression. These data and analyses raise the question of whether the TCR CDR3-DDX53 chemical complementarity assessment detected an ESCA immune response that selected for DDX53-negative cells?

## INTRODUCTION

Esophageal cancer is the eighth most common cancer and the sixth leading cause of cancer death worldwide [1]. Esophageal squamous cell carcinoma (ESCC) is the predominant histological type and comprises 90% of cases [2], with most of the remaining cases representing esophageal adenocarcinoma. The five-year survival for esophageal cancer remains unfavorable, at 15–25%, despite advancements in early endoscopic detection and multidisciplinary intervention [1, 2]. The presence of an immune response mediated by tumor-infiltrating lymphocytes, however, has been shown to be strongly associated with longer disease-free survival (DFS) in some esophageal cancer patients [2, 3]. Due to their high *in vivo* immunogenicity and elevated expression in primary esophageal cancer specimens, cancer-testis antigens (CTAs) in particular are under investigation as potential targets for T-lymphocyte based esophageal cancer immunotherapy [4].

On the other hand, while a targeted, T-cell mediated immune response may improve outcomes in some esophageal cancer patients, in a comparatively larger number of patients, T-cell recruitment and their subsequent activation of pro-tumorigenic immune cell populations has been shown to associate with overall poorer prognosis in ESCC [5]. T-lymphocytes are known to recruit and activate tumor-associated macrophages (TAMs) of the M2 pro-inflammatory phenotype to the tumor microenvironment. M2 TAMs subsequently expand myeloid-derived suppressor cells (MDSCs) through the IL-6 pathway, inducing a pro-tumorigenic microenvironment. In ESCC patients, M2 TAM accumulation and high serum levels of IL-6 are both demonstrably present [6].

To better understand the potential positive and negative impacts of T-cells in ESCA, we recovered TCR recombination reads from the TCGA-ESCA whole exome sequence (WXS) files, obtained the TCR CDR3 AA sequences, and assessed the chemical complementarity of those sequences with CTAs, as those antigens have been characterized by many approaches over decades as candidate cancer antigens. Results indicated that a high level of chemical complementarity between tumor resident, TCR CDR3s and the CTA, DDX53, was associated with a worse outcome, in contrast to several previous assessments whereby immune receptor CDR3-candidate antigen chemical complementarities were associated with better outcomes [7–12]. In this ESCA study, the possibility of an immune response that selected for tumor cells lacking the DDX53 CTA is discussed.

## RESULTS

### TCR CDR3-CTA chemical complementarities associated with reduced DFS probabilities

**Figure 1:**
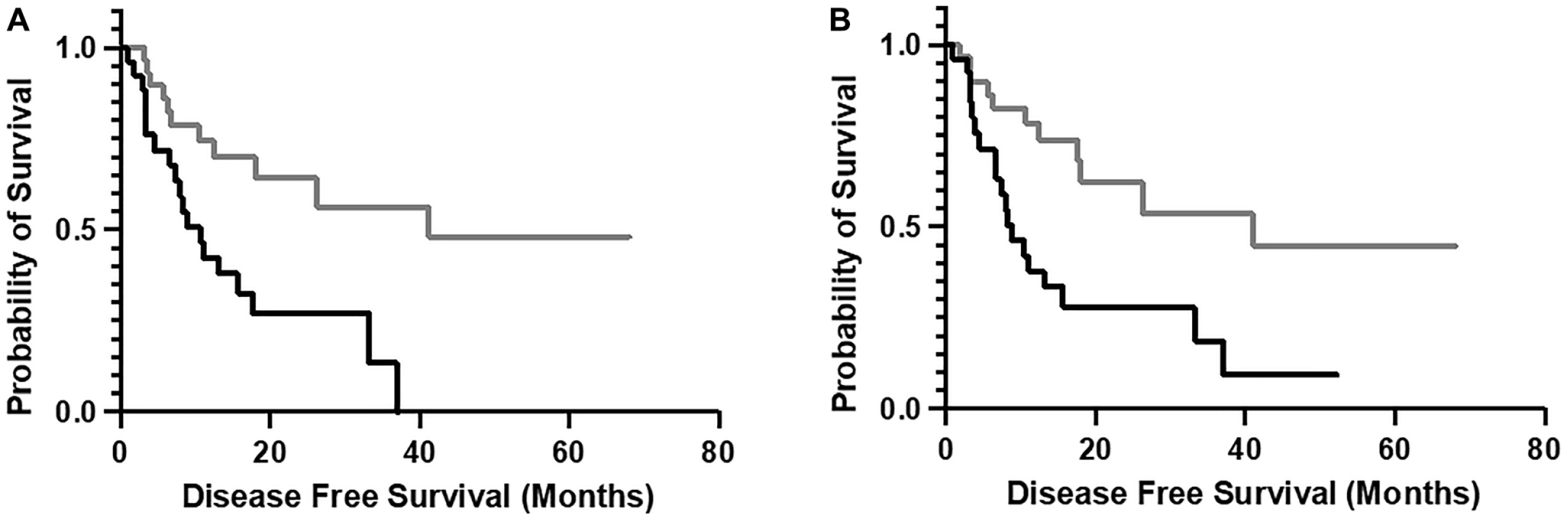
Disease-free survival (DFS) distinction associated with Combo complementarity scores (CS) for the cancer testis antigen, DDX53, and tumor resident TCR-CDR3s. (**A**) Full length DDX53; black line, upper 50th percentile complementarity group (median DFS, 10.68 months); gray line, lower 50th percentile complementarity group (median DFS, 41.13 months). Logrank *p*-value = 0.003; Cox regression *p*-value = 0.012. (**B**) DDX53 peptide fragment (AA sequence: MNNSVNLRSITYLVIDEADKMLDMEFEPQIRKILLDVRPDRQTVMTSATWPDTVRQLALS). Black line, upper fiftieth percentile complementarity group (median DFS, 14.29 months); gray line, lower fiftieth percentile complementarity group (median DFS, 32.42). Logrank *p*-value = 0.005. Cox regression *p*-value = 0.016. The y-axis represents survival probability.

As noted in Introduction, recent work has indicated opportunities to stratify cancer cases based on the chemical complementarities of the adaptive IR CDR3 AA sequences and candidate cancer antigens, using algorithms particularly designed for a big data setting [7–11, 13] (Methods). Thus, we recovered TRA and TRB recombination reads from the tumor WXS files representing the TCGA-ESCA dataset (Supplementary Table 1), translated the CDR3s represented by productive recombinations, and specifically applied the algorithm of ref. [13] to determine whether the chemical complementarity between the TCGA-ESCA TCR CDR3s and a set of CTAs [14] also represented DFS distinctions. Results indicated an inverse association between Combo complementarity scores (CSs) (Methods) and DFS. Specifically, the upper 50th percentile, tumor-based, TCR CDR3-DDX53 Combo CSs correlated with a lower DFS (Figure 1A). This inverse survival association was also observed for the Combo CSs for the TCR CDR3s and a DDX53 peptide fragment (AA sequence: MNNSVNLRSITYLVIDEADKMLDMEFEPQIRKILLDVRPDRQTVMT SATWPDTVRQLALS) (Figure 1B).

### Reduced DDX53 expression in ESCA specimens representing high TCR CDR3-DDX53 Combo CSs

To determine whether DDX53 expression was reduced in ESCA specimens with higher TCR CDR3-DDX53 Combo CSs, we conducted an odds-ratio analysis for “zero DDX53 expression” in the tumor specimens representing the upper and lower 50th percentile TCR CDR3-DDX53 Combo CS groups. Results indicated that the upper 50th percentile Combo CS group represented a greater proportion of ESCA specimens with no expression of DDX53 (Table 1).

**Table 1:** A higher number of samples with zero DDX53 expression in the high TCR CDR3-DDX53 Combo CS group

Combo CS group	Proportion with zero expression	*p*-value
Upper fiftieth percentile	82.9%	0.052
Lower fiftieth percentile	62.2%	

### Increased DNA methylation of DDX53 in ESCA specimens representing the upper 50th percentile of the TCR CDR3-DDX53 Combo CS group

To consider a mechanism of reduced DDX53 expression in the ESCA specimens with higher TCR CDR3-DDX53 Combo complementarity, we conducted a methylation analysis of the tumor specimens belonging to the upper and lower 50th percentile Combo CS groups. Results indicated that methylation of the DDX53 gene was comparatively increased in the upper 50th percentile Combo CS group (Table 2), although the statistical analysis represented the standard of a trend rather than significance.

**Table 2:** Increased methylation of the DDX53 gene in the high TCR CDR3-DDX53 Combo CS group

Combo CS group	Mean β-value	*p*-value
Upper fiftieth percentile	0.92	0.083
Lower fiftieth percentile	0.87	

### EIF2AK3 and POLG expression levels correlated with the TCR CDR3-DDX53 Combo CSs

**Figure 2:**
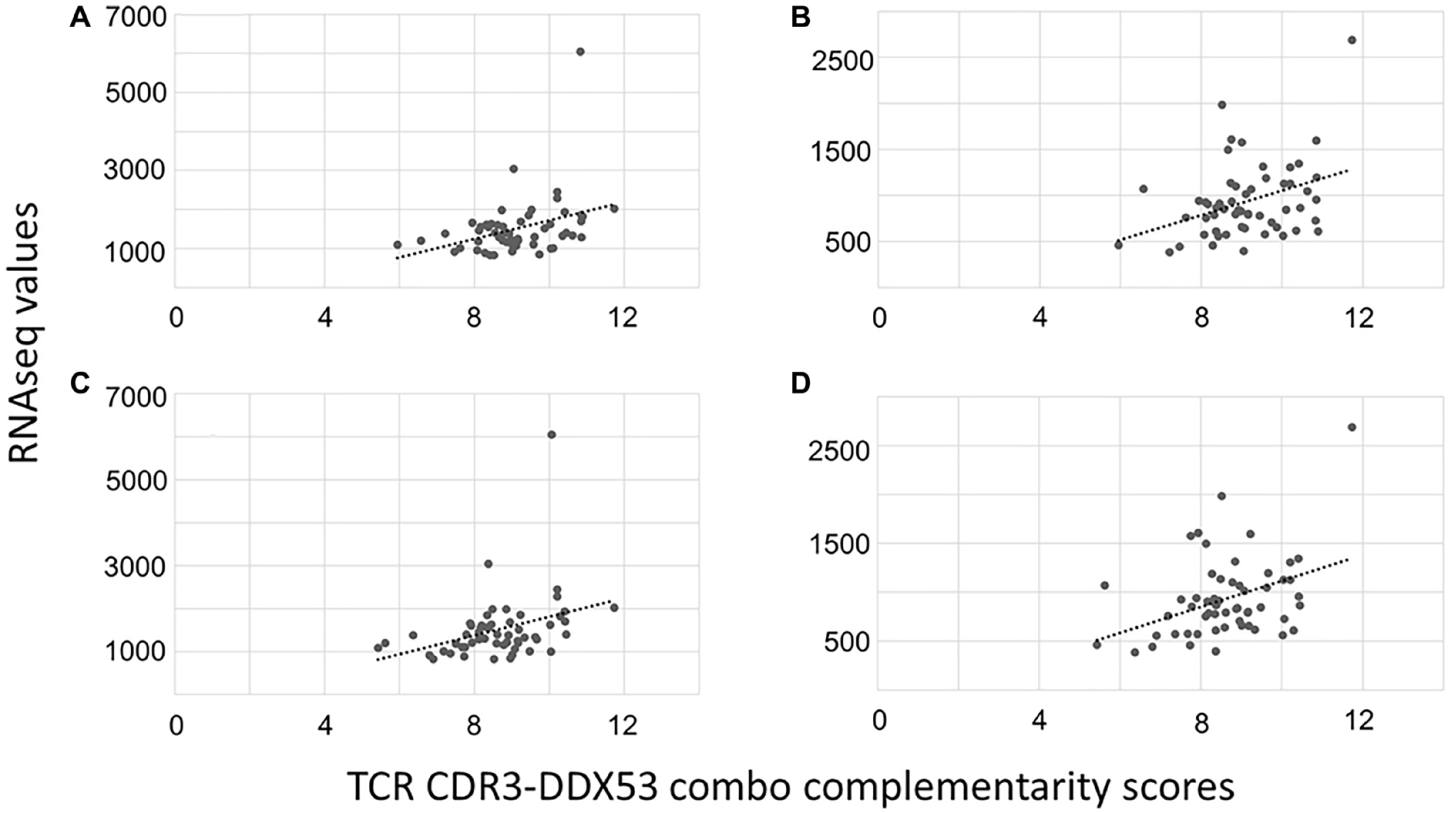
Expression of proliferation markers POLG and EIF2AK3 positively correlate with TCR CDR3-DDX53 Combo CSs. (**A**) POLG expression, positive correlation with CSs for the TCR CDR3-DDX53 pair. Pearson coefficient = 0.36, *p*-value = 0.004. (**B**) EIF2AK3 expression, positive correlation with CSs for the TCR CDR3-DDX53 pair. Pearson coefficient = 0.37, *p*-value = 0.005. (**C**) POLG expression correlated with CSs for the TCR CDR3-DDX53 peptide fragment pair (peptide AA sequence: MNNSVNLRSITYLVIDEADKMLDMEFEPQIRKILLDVRPDRQTVMTSATWPDTVRQLALS). Pearson coefficient = 0.35, *p*-value = 0.008. (**D**) EIF2AK3 expression correlated with CSs for the TCR CDR3-DDX53 peptide fragment pair. Pearson coefficient = 0.39, *p*-value = 0.003.

To determine whether pro-proliferative molecular markers correlated with higher TCR CDR3-DDX53 Combo CSs, due to the correlation of the higher TCR CDR3-DDX53 Combo CSs and worse DFS, we evaluated the RNA-seq values of a series of pro-proliferative effector genes [15] for the tumor specimens corresponding to the CS calculations. Results indicated that EIF2AK3 and POLG expression levels correlated with the TCR CDR3-DDX53 Combo CSs, consistent with a higher proliferation rate for the tumor cells representing the higher CSs and worse DFS probabilities (Figure 2). However, an evaluation of the entire TCGA-ESCA dataset, for a survival distinction based on the expression levels of these two genes, did not reveal a survival distinction (data not shown), consistent with a mechanistic specificity of the TCR CDR3-DDX53 interaction reflecting a worse DFS probability.

### DDX53 Combo CS is a DFS marker independent of several other ESCA clinical features

To determine whether the survival distinction represented by DDX53 Combo CSs was independent of clinical variables known to distinguish ESCA outcomes, we performed bivariate analyses of DDX53 Combo complementarity against several of these variables. (There were too few samples for a combined, multivariate analysis.) Results indicated that, in each bivariate comparison, DDX53 Combo CSs remained significant in representing a worse DFS (Table 3).

**Table 3:** Bivariate analysis of TCR CDR3-DDX53 Combo CSs and several ESCA clinical features

Comparison	Exp(β)	*p*-value
CDR3-DDX53 Combo CS Race	3.41 2.00	0.006 0.100
CDR3-DDX53 Combo CS Sex	2.98 2.23	0.006 0.270
CDR3-DDX53 Combo CS Alcohol Consumption	4.54 1.20	0.009 0.057
CDR3-DDX53 Combo CS Neoplasm Histological Grade	2.57 2.27	0.020 0.267
CDR3-DDX53 Combo CS Columnar Metaplasia Present	3.77 1.30	0.019 0.604
CDR3-DDX53 Combo CS Fraction of Genome Altered	2.75 6.89	0.011 0.050

The analyses assessed the correlation of the reference value with hazard, and thus an exponent > 1.0 represents a positive correlation with hazard. Where the *p*-value is above 0.05, there is no accepted statistical significance of association with DFS. However, in every case, the indicated clinical feature, as well as the CDR3-DDX3 CSs, was significantly associated with DFS in univariate analyses.

## DISCUSSION

We observed TCR CDR3-DDX53 chemical complementarity inversely (negatively) associating with ESCA DFS, with the potential TCR-DDX53 interaction representing tumor resident, TCR CDR3 AA sequences. Most importantly, tumor specimens with higher TCR CDR3-DDX53 CSs were less likely to express DDX53, consistent with the possibility that the TCR-DDX53 interaction selected against tumor cells expressing DDX53. Additionally, the tumor specimens with higher TCR CDR3-DDX53 CSs represented greater DDX53 gene methylation, consistent with the lower expression level of DDX53 among the high CS samples and also suggesting a mechanism for the loss of DDX53 expression. Overall, these data are consistent with the possibility that TCR-antigen interaction induces selective pressure that results in antigen loss, a process referred to as immuno-editing [16–18], and, eventually, tumor cell escape from an anti-tumor immune response. These data for ESCA are also consistent with similar data obtained for high CS, low survival in the ovarian cancer setting [19].

At the point when CTAs are no longer present, the sustained involvement of T-cells may induce pro-inflammatory, pro-tumorigenic effects through general features of the immune response. In ESCC, it is likely that this process occurs in large part due to the accumulation of M2 TAMs [6]. Further, poor ESCC prognosis is known to associate with the activation and accumulation of such tumorigenic immune cells [5], lending credence to our inference that a vestigial immune response could be deleterious across ESCA subtypes. Finally, if DDX53 expression is selected against in early ESCA tumors, then an assay for DDX53 may be useful as an early screening tool for ESCA.

Noting several limitations of this study, it would be important to attempt to repeat the results using an immune repertoire, PCR-based approach to obtaining tumor TCR recombination reads, to have a more comprehensive collection of productive CDR3s. And, the tentative conclusions here may be further supported with a prospective clinical trial, allowing for a better management of potentially confounding variables.

## METHODS

### Recovery of the TCGA-ESCA T-cell receptor (TCR) recombination reads

The recovery of the TRA and TRB recombination reads from the TCGA-ESCA exome (WXS) files was performed as described [20–22]. Briefly, the WXS files were searched with a low stringency match to all TCR V- and J-gene segments. The reads in those output files were then searched for a validated V- and J-gene segment on one read, and only reads with a productive complementarity determining region-3 (CDR3) amino acid (AA) sequence were used in this study, i.e., reads with V-J joins that did not have a stop codon or an out-of-frame junction. The original software pipeline used for the extraction of the recombination reads from the WXS files, for this report, is freely available at https://github.com/bchobrut-USF/blanck_group, which includes a readme file; and a containerized version of the code is freely available at https://hub.docker.com/r/bchobrut/vdj, also with a readme file. In addition, an updated version with some refinements is available at https://github.com/kcios/2021. The entire collection of TCR recombination read output data used in this report is available in the supporting online material (SOM) Supplementary Table 1. The WXS files were accessed via NIH dbGaP project approval number 6300. Finally, basic benchmarking of the recovery of adaptive immune receptor recombination reads from genomics files can be reviewed in the following refs. [23–27].

### Chemical complementarity scoring

The chemical complementarity scoring for the CDR3-cancer testis antigen combinations was performed as described [13], and can be conducted with a publicly available, original web tool, https://adaptivematch.com/. The web tool includes instructions, and example input files, used in this report, are available in the SOM, Supplementary Tables 2–4. (However, to use Supplementary Tables 2–4 as input for the web tool, those files will have to be re-saved by the user as csv files.) The web tool has been extensively benchmarked in refs. [28, 29], and further employed in refs [30, 31]. Supplementary Table 2 represents CDR3s, Supplementary Table 3 represents CTAs, and Supplementary Table 4 represents DFS data publicly available at cbioportal.org. Briefly, the scoring process involves an alignment of a CDR3-CTA, a calculation of the chemical attractiveness based on the aligned CDR3-CTA, and a one-AA shift, and a re-calculation. The details of the calculation process are in ref [13], but briefly, two AA directly opposite of each other in the alignment, with opposite electrostatic charges would contribute to a high chemical complementarity score, and if those AAs are shifted by one position, the value of their contribution to the score is reduced. A similar standard is applied to a hydrophobicity version of the AA chemical attractiveness, based on Uversky hydropathy values for each AA [11], whereby higher Uversky hydropathy values represent higher hydrophobicity. The best CS is retained for any given CDR3-CTA assessment. A “Combo CS” represents a CS calculation that integrates both electrostatic and Uversky hydropathy AA values. SOM Supplementary Tables 5 and 6 represent the https://adaptivematch.com/ web tool output for SOM Supplementary Tables 2–4 and represent the results of the chemical complementarity scoring algorithm developed in ref [13]. These results allow spot checking of the high and low scores, for example, for electrostatic attractiveness, by examining the alignment in the output (Supplementary Table 6) that represented the best score for a given electrostatic, CDR3-CTA matchup.

### DNA methylation analysis of DDX53

DNA methylation array-based beta values representing the extent of site-specific gene methylation were obtained from datasets available at https://gdc.cancer.gov/. The distinction of the DDX53 methylation values for the upper and lower 50th percentile TCR CDR3-DDX53 Combo CSs was evaluated using a two-tailed Student’s *T*-test (Supplementary Table 7).

### Gene expression and follow up, pro-proliferation biomarkers

The expression levels of various genes were assessed with RSEM values from an RNA-seq based dataset available at cbioportal.org. A distinction of the percentage of tumor samples representing zero DDX53 expression in the upper and lower 50th percentile TCR CDR3-DDX53 Combo CSs was evaluated using an odds-ratio test (Supplementary Table 8). Correlation between expression of pro-proliferation biomarkers and TCR CDR3-DDX53 Combo CSs was evaluated using a Pearson’s correlation as further detailed in Results.

### Multivariate analysis

A multivariate analysis was conducted using the IBM Statistical package for the social sciences (SPSS), recently renamed as, Statistical Product and Service Solutions. TCR CDR3-DDX53 Combo CSs were compared against patient race, sex, frequency of alcohol consumption, neoplasm histologic grade, presence of columnar metaplasia, and genome alteration fraction, as further detailed in Results.

## SUPPLEMENTARY MATERIALS



















## Abbreviations

AA: amino acid
CDR3: complementarity determining region-3
CS: complementarity score
CTA: cancer testis antigen
dbGaP: database of Genotypes and Phenotypes
DFS: disease-free survival
ESCA: esophageal cancer (TCGA abbreviation for both ESCC and esophageal adenocarcinoma)
ESCC: esophageal squamous cell carcinoma
GDC: genomic data commons
HUGO: human genome organization
IR: (adaptive) immune receptor
KM: Kaplan-Meier
MDSC: myeloid-derived suppressor cells
NIH: National Institutes of Health
PCR: polymerase chain reaction
TAM: tumor-associated macrophage
TCGA: the cancer genome atlas
TCR: T-cell receptor
TRA: HUGO symbol for T-cell receptor alpha gene
TRB: HUGO symbol for T-cell receptor beta gene
WXS: whole exome sequence

## References

[1] Domper Arnal MJ, Ferrández Arenas Á, Lanas Arbeloa Á. Esophageal cancer: Risk factors, screening and endoscopic treatment in Western and Eastern countries. World J Gastroenterol. 2015; 21:7933–43. 10.3748/wjg.v21.i26.7933. 26185366 PMC4499337

[2] Baba Y, Nomoto D, Okadome K, Ishimoto T, Iwatsuki M, Miyamoto Y, Yoshida N, Baba H. Tumor immune microenvironment and immune checkpoint inhibitors in esophageal squamous cell carcinoma. Cancer Sci. 2020; 111:3132–41. 10.1111/cas.14541. 32579769 PMC7469863

[3] Yagi T, Baba Y, Ishimoto T, Iwatsuki M, Miyamoto Y, Yoshida N, Watanabe M, Baba H. PD-L1 Expression, Tumor-infiltrating Lymphocytes, and Clinical Outcome in Patients With Surgically Resected Esophageal Cancer. Ann Surg. 2019; 269:471–78. 10.1097/SLA.0000000000002616. 29206673

[4] Zhang Y, Zhang Y, Zhang L. Expression of cancer-testis antigens in esophageal cancer and their progress in immunotherapy. J Cancer Res Clin Oncol. 2019; 145:281–91. 10.1007/s00432-019-02840-3. 30656409 PMC6373256

[5] Bhat AA, Nisar S, Maacha S, Carneiro-Lobo TC, Akhtar S, Siveen KS, Wani NA, Rizwan A, Bagga P, Singh M, Reddy R, Uddin S, Grivel JC, et al. Cytokine-chemokine network driven metastasis in esophageal cancer; promising avenue for targeted therapy. Mol Cancer. 2021; 20:2. 10.1186/s12943-020-01294-3. 33390169 PMC7780621

[6] Lin EW, Karakasheva TA, Hicks PD, Bass AJ, Rustgi AK. The tumor microenvironment in esophageal cancer. Oncogene. 2016; 35:5337–49. 10.1038/onc.2016.34. 26923327 PMC5003768

[7] Yeagley M, Chobrutskiy BI, Gozlan EC, Medikonda N, Patel DN, Falasiri S, Callahan BM, Huda T, Blanck G. Electrostatic Complementarity of T-Cell Receptor-Alpha CDR3 Domains and Mutant Amino Acids Is Associated with Better Survival Rates for Sarcomas. Pediatr Hematol Oncol. 2021; 38:251–64. 10.1080/08880018.2020.1843576. 33616477

[8] Hsiang M, Chobrutskiy BI, Diaz M, Huda TI, Creadore S, Zaman S, Cios KJ, Gozlan EC, Blanck G. Chemical complementarity between immune receptors and cancer mutants, independent of antigen presentation protein binding, is associated with increased survival rates. Transl Oncol. 2021; 14:101069. 10.1016/j.tranon.2021.101069. 33780706 PMC8039726

[9] Arturo JF, Chobrutskiy BI, Yeagley M, Patel DN, Falasiri S, Patel JS, Blanck G. Electrostatic complementarity of B-cell receptor CDR3s and TP53-mutant amino acids in breast cancer is associated with increased disease-free survival rates. Cell Mol Immunol. 2020; 17:776–78. 10.1038/s41423-019-0328-8. 31729463 PMC7331692

[10] Chobrutskiy BI, Yeagley M, Diviney A, Zaman S, Gozlan EC, Tipping P, Koohestani DM, Roca AM, Blanck G. A scoring system for the electrostatic complementarities of T-cell receptors and cancer-mutant amino acids: multi-cancer analyses of associated survival rates. Immunology. 2020; 159:373–83. 10.1111/imm.13165. 31821535 PMC7077996

[11] Chobrutskiy BI, Yeagley M, Tipping P, Zaman S, Diviney A, Patel DN, Falasiri S, Uversky VN, Blanck G. Chemical complementarity between immune receptor CDR3s and IDH1 mutants correlates with increased survival for lower grade glioma. Oncogene. 2020; 39:1773–83. 10.1038/s41388-019-1101-2. 31740784

[12] Chobrutskiy BI, Zaman S, Diviney A, Mihyu MM, Blanck G. T-cell receptor-α CDR3 domain chemical features correlate with survival rates in bladder cancer. J Cancer Res Clin Oncol. 2019; 145:615–23. 10.1007/s00432-018-2815-1. 30539280 PMC11810338

[13] Chobrutskiy BI, Chobrutskiy A, Zaman S, Yeagley M, Huda TI, Blanck G. High-throughput, sliding-window algorithm for assessing chemical complementarity between immune receptor CDR3 domains and cancer mutant peptides: TRG-PIK3CA interactions and breast cancer. Mol Immunol. 2021; 135:247–53. 10.1016/j.molimm.2021.02.026. 33933816

[14] Almeida LG, Sakabe NJ, deOliveira AR, Silva MC, Mundstein AS, Cohen T, Chen YT, Chua R, Gurung S, Gnjatic S, Jungbluth AA, Caballero OL, Bairoch A, et al. CTdatabase: a knowledge-base of high-throughput and curated data on cancer-testis antigens. Nucleic Acids Res. 2009; 37:D816–19. 10.1093/nar/gkn673. 18838390 PMC2686577

[15] Mauro JA, Yavorski JM, Blanck G. Stratifying melanoma and breast cancer TCGA datasets on the basis of the CNV of transcription factor binding sites common to proliferation- and apoptosis-effector genes. Gene. 2017; 614:37–48. 10.1016/j.gene.2017.02.026. 28257835

[16] Ostrand-Rosenberg S. Immune surveillance: a balance between protumor and antitumor immunity. Curr Opin Genet Dev. 2008; 18:11–18. 10.1016/j.gde.2007.12.007. 18308558 PMC2699403

[17] Ikeda H, Old LJ, Schreiber RD. The roles of IFN gamma in protection against tumor development and cancer immunoediting. Cytokine Growth Factor Rev. 2002; 13:95–109. 10.1016/s1359-6101(01)00038-7. 11900986

[18] Martin TD, Patel RS, Cook DR, Choi MY, Patil A, Liang AC, Li MZ, Haigis KM, Elledge SJ. The adaptive immune system is a major driver of selection for tumor suppressor gene inactivation. Science. 2021; 373:1327–35. 10.1126/science.abg5784. 34529489 PMC13397623

[19] Barker VR, Varkhedi M, Patel DN, Hsiang M, Chobrutskiy A, Chobrutskiy BI, Blanck G. TCR CDR3-antigen chemical complementarity associated with poor ovarian cancer outcomes: A vestigial immune response to early cancer antigens? Am J Reprod Immunol. 2023; 89:e13639. 10.1111/aji.13639. 36317868

[20] Gill TR, Samy MD, Butler SN, Mauro JA, Sexton WJ, Blanck G. Detection of Productively Rearranged TcR-α V-J Sequences in TCGA Exome Files: Implications for Tumor Immunoscoring and Recovery of Antitumor T-cells. Cancer Inform. 2016; 15:23–28. 10.4137/CIN.S35784. 26966347 PMC4768948

[21] Tong WL, Tu YN, Samy MD, Sexton WJ, Blanck G. Identification of immunoglobulin V(D)J recombinations in solid tumor specimen exome files: Evidence for high level B-cell infiltrates in breast cancer. Hum Vaccin Immunother. 2017; 13:501–6. 10.1080/21645515.2016.1246095. 28085544 PMC5360147

[22] Chobrutskiy BI, Zaman S, Tong WL, Diviney A, Blanck G. Recovery of T-cell receptor V(D)J recombination reads from lower grade glioma exome files correlates with reduced survival and advanced cancer grade. J Neurooncol. 2018; 140:697–704. 10.1007/s11060-018-03001-1. 30382482

[23] Thorsson V, Gibbs DL, Brown SD, Wolf D, Bortone DS, Ou Yang TH, Porta-Pardo E, Gao GF, Plaisier CL, Eddy JA, Ziv E, Culhane AC, Paull EO, et al. The Immune Landscape of Cancer. Immunity. 2018; 48:812–30.e14. 10.1016/j.immuni.2018.03.023. 29628290 PMC5982584

[24] Li B, Li T, Wang B, Dou R, Zhang J, Liu JS, Liu XS. Ultrasensitive detection of TCR hypervariable-region sequences in solid-tissue RNA-seq data. Nat Genet. 2017; 49:482–83. 10.1038/ng.3820. 28358132 PMC6959004

[25] Patel DN, Yeagley M, Arturo JF, Falasiri S, Chobrutskiy BI, Gozlan EC, Blanck G. A comparison of immune receptor recombination databases sourced from tumour exome or RNAseq files: Verifications of immunological distinctions between primary and metastatic melanoma. Int J Immunogenet. 2021; 48:409–18. 10.1111/iji.12550. 34298587

[26] Clark KR, Tong WL, Callahan BM, Yavorski JM, Tu YN, Blanck G. TRB-J1 usage, in combination with the HLA-A*01:01 allele, represents an apparent survival advantage for uterine corpus endometrial carcinoma: Comparisons with microscopic assessments of lymphocyte infiltrates. Int J Immunogenet. 2019; 46:31–37. 10.1111/iji.12409. 30474304

[27] Brown SD, Raeburn LA, Holt RA. Profiling tissue-resident T cell repertoires by RNA sequencing. Genome Med. 2015; 7:125. 10.1186/s13073-015-0248-x. 26620832 PMC4666197

[28] Eakins RA, Chobrutskiy A, Teer JK, Patel DN, Hsiang M, Huda TI, Zaman S, Sexton WJ, Coppola D, Falasiri S, Blanck G, Chobrutskiy BI. Chemical complementarity between tumor resident, T-cell receptor CDR3s and MAGEA3/6 correlates with increased melanoma survival: Potential relevance to MAGE vaccine auto-reactivity. Mol Immunol. 2022; 150:58–66. 10.1016/j.molimm.2022.08.001. 35987136

[29] Pakasticali N, Chobrutskiy A, Patel DN, Hsiang M, Zaman S, Cios KJ, Blanck G, Chobrutskiy BI. Chemical Complementarity of Breast Cancer Resident, T-Cell Receptor CDR3 Domains and the Cancer Antigen, ARMC3, is Associated With Higher Levels of Survival and Granzyme Expression. Cancer Inform. 2023; 22:11769351231177269. 10.1177/11769351231177269. 37313373 PMC10259117

[30] Patel AR, Patel DN, Tu YN, Yeagley M, Chobrutskiy A, Chobrutskiy BI, Blanck G. Chemical complementarity between immune receptor CDR3s and candidate cancer antigens correlating with reduced survival: evidence for outcome mitigation with corticosteroid treatments. J Biomol Struct Dyn. 2023; 41:4632–40. 10.1080/07391102.2022.2070546. 35538689

[31] Huda TI, Diaz MJ, Gozlan EC, Chobrutskiy A, Chobrutskiy BI, Blanck G. Immunogenomics Parameters for Patient Stratification in Alzheimer’s Disease. J Alzheimers Dis. 2022; 88:619–29. 10.3233/JAD-220119. 35662120

