# Supplementary material for: An immunoinformatics assessment of the cancer testis antigen, DDX53, as a potential early esophageal cancer antigen
Source: Oncoscience. 2023 Nov 10;10:59–66. doi: 10.18632/oncoscience.590 (PMC10637345; doi:10.18632/oncoscience.590)
Supplement: Supplementary file 1 [file oncoscience-10-590-s001.pdf]

An immunoinformatics assessment of the cancer testis antigen, DDX53, as a potential early esophageal cancer antigen

SUPPLEMENTARY MATERIALS

Supplementary Table 1: Adaptive immune receptor recombination read data obtained from mining the TCGA-ESCA WXS files (including single value, physicochemical values representing the CDR3 AA sequences, on a separate Excel file sheet). See Supplementary Table 1

Supplementary Table 2: TRA TRB ESCA tumor CDR3, input file for http://adaptivematch.com. See Supplementary Table 2

Supplementary Table 3: antigens ddx53, input file for http://adaptivematch.com. See Supplementary Table 3

Supplementary Table 4: ESCA DFS survival data, input file for http://adaptivematch.com. See Supplementary Table 4

Supplementary Table 5: Adaptive match output file, summary. See Supplementary Table 5

Supplementary Table 6: Adaptive match output file, chemical complementarity scores. See Supplementary Table 6

Supplementary Table 7: DNA methylation values for DDX53, upper and lower 50th percentile, TCR CDR3-DDX53 Combo CS groups. See Supplementary Table 7

Supplementary Table 8: Gene expression values for DDX53, upper and lower 50th percentile, TCR CDR3-DDX53 Combo CS groups. See Supplementary Table 8
